# Serum Tryptophan and Kynurenine/Tryptophan Ratio as Markers of Depressive Symptoms in Chronic Obstructive Pulmonary Disease (COPD)

**DOI:** 10.7759/cureus.95267

**Published:** 2025-10-23

**Authors:** Anli Liu, Zhong Li, Gang Yao, Yuqin Kuang, Bei Guo, Rongzhi Zhang, Huirong Li, Chunyan Xie

**Affiliations:** 1 Department of Respiratory and Critical Care Medicine, The First People's Hospital of Longquanyi District, Chengdu, CHN; 2 Department of Psychology, The First People's Hospital of Longquanyi District, Chengdu, CHN; 3 Department of Acute Psychiatry, The Fourth People's Hospital of Chengdu, Chengdu, CHN; 4 Department of Gastroenterology, The First People's Hospital of Longquanyi District, Chengdu, CHN

**Keywords:** chronic obstructive pulmonary disease, depression, kynurenine, prospective cross-sectional case-control observational study, tryptophan

## Abstract

Background and aim

Depression is a common comorbidity in patients with chronic obstructive pulmonary disease (COPD). Given the involvement of the tryptophan-kynurenine pathway in depression, this study aimed to investigate the potential clinical value of the serum markers of tryptophan-kynurenine metabolism in COPD-related depressive symptoms.

Methods

A comparative cross-sectional study was designed. The serum levels of tryptophan and kynurenine were measured by liquid chromatography-mass spectrometry in consecutive adult patients with severe COPD. The tryptophan, kynurenine, and kynurenine-to-tryptophan ratio (KTR) were investigated as potential markers for depressive symptoms, which were classified by the six-item Hamilton Depression subscale (HDS-6). The associations of tryptophan-kynurenine pathway markers with depressive symptoms were assessed using Spearman's correlation and logistic regression models.

Results

Among the included 102 patients with COPD, 44 (43.1%) had depressive symptoms. Compared to the controls, the patients with depressive symptoms had lower serum levels of tryptophan and a higher kynurenine-to-tryptophan ratio (KTR). The severity of depressive symptoms, as measured by the HDS-6 score, showed a significant negative correlation with tryptophan levels (ρ=-0.478, p<0.001) and a positive correlation with KTR (ρ=0.613, p<0.001). In multivariate logistic analyses, higher serum tryptophan quartiles were associated with a decreasing risk of depressive symptoms, while higher KTR quartiles were associated with an increasing risk (p for trend <0.001 for both).

Conclusion

In this cohort of COPD patients, lower serum tryptophan and a higher kynurenine-to-tryptophan ratio (KTR) were significantly associated with the presence and severity of depressive symptoms. These findings nominate serum tryptophan and KTR as potential biomarkers for assessing depressive symptoms in COPD, which could improve diagnostic accuracy and guide treatment strategies.

## Introduction

Chronic obstructive pulmonary disease (COPD) is a common chronic respiratory disease characterized by progressive and persistent airflow limitation [1]. Although the mortality and disability rates of COPD have been gradually decreasing, its incidence has been slowly rising over the past 20 years, and it remains an important public health problem [2]. Mental health-related disorders, including depression, are important comorbidities of COPD [3]. Among hospitalized patients with acute exacerbations of COPD, 67.7% were diagnosed with probable depression, and depression was established in 41.7% [4]. The comorbid depression impairs quality of life and reduces adherence, which contribute to a substantial COPD-related burden [3]. The biological mechanisms of depression are complex and have not been fully elucidated at present [5,6]. Thus, traditional antidepressant medication remains the primary method for treating this psychological disorder, but its efficacy is not entirely satisfactory [5]. In addition, there is still a lack of biomarkers for identifying and assessing this type of depression [7].

Serotonin, a key neurotransmitter involved in various behavioral and cognitive functions, has long been implicated in depression [8]. However, a comprehensive review of existing evidence suggests that the relationship between reduced serotonin levels and depression is not as straightforward as previously thought [5]. This has led researchers to focus on tryptophan, the precursor of serotonin [7]. The kynurenine pathway is the predominant route of tryptophan catabolism, accounting for over 95% of its metabolism [9]. This pathway involves several key enzymes, including tryptophan-2,3-dioxygenase, indoleamine-2,3-dioxygenase (IDO) 1, and IDO 2 [9]. These enzymes catalyze the metabolism of tryptophan along the kynurenine pathway, which can then be further metabolized into various neuroactive compounds, such as kynurenic acid, quinolinic acid, and 3-hydroxykynurenine, ultimately resulting in the formation of kynurenine [6,9]. Extensively studied in the context of immune responses and neuroinflammation, the kynurenine pathway has been shown to produce neurotoxic and neuroprotective metabolites upon activation, which may contribute to the development of depression [9]. For instance, kynurenic acid acts as a natural antagonist of many glutamate receptors and has been implicated in the pathophysiology of depression [9]. Additionally, increased levels of quinolinic acid have been observed in the cerebrospinal fluid of patients with major depressive disorder [9]. Particularly, tryptophan and kynurenine are more commonly used in research as they reflect the entire metabolic axis and have been more extensively studied in the context of depression. A meta-analysis of 101 studies showed that the kynurenine-to-tryptophan ratio (KTR) is increased in major depressive disorder [10]. Similar findings have been observed in patients with inflammatory bowel disease, where imbalanced tryptophan-kynurenine metabolism is associated with depression [11].

In the context of COPD, hypoxia and systemic inflammation are well-established pathophysiological features [12,13]. Furthermore, both hypoxia and inflammation have been independently shown to activate the kynurenine pathway of tryptophan metabolism, which is a proposed mechanism contributing to the development of depressive symptoms [14,15]. Therefore, tryptophan metabolites along the kynurenine pathway may serve as potential markers for COPD-related depression.

This study aimed to determine the associations between serum tryptophan, kynurenine, and the kynurenine-to-tryptophan ratio (KTR) with the presence and severity of depressive symptoms in patients with severe COPD.

## Materials and methods

Study design and definitions

We conducted this comparative cross-sectional study on COPD-related depressive symptoms from January 2023 to March 2023. The COPD was diagnosed and graded based on clinical symptoms, signs, history of exposure to risk factors, and spirometry results, according to the Global Initiative for Chronic Obstructive Lung Disease (GOLD) criteria [1]. The GOLD criteria define COPD as a post-bronchodilator ratio of forced expiratory volume in the first second to forced vital capacity (FEV1/FVC) of less than 0.70. GOLD stages C or D were defined as severe COPD.

Depressive symptom was classified by the six-item Hamilton Depression subscale (HDS-6), a simplified version of the 17-item Hamilton Depression scale (HDS-17) [16]. The HDS-6 assesses core depressive symptoms across six items as follows: depressed mood, feelings of guilt, suicide, work and activities (insomnia-middle), psychomotor retardation, and psychic anxiety. Each item is scored on a scale of 0-2 or 0-4, with higher scores indicating greater severity. Previous studies have confirmed the superior internal validity of the HDS-6 compared to the HDS-17 in COPD populations [16]. The cut-off score for depressive symptoms was set to 7 or more, and for major depression was 10 or more in this study, as described previously [16]. Throughout this study, COPD patients with an HDS-6 score below 7 were designated as controls, while those with an HDS-6 score of 7-9 were classified as having non-major depressive symptoms. The HDS-6 was administered by ZL, a trained clinical psychologist who was blinded to the patients' clinical status regarding COPD and their serum tryptophan and kynurenine levels.

Subjects

A consecutive sampling technique was employed to recruit adult patients with severe COPD (GOLD stages C or D). The key inclusion criteria were clinical stability and age between 18 and 85 years. Patients with the following conditions were excluded: (1) unstable clinical conditions, or dysfunctions of any organ, including lung, heart, kidney, and liver; (2) acute exacerbation of COPD; (3) pregnant or delivery; (4) any malignant disease; (5) any active infection; (6) glucocorticoid treatment within one month; (7) cognition impairment; (8) alcohol or drug abuse; (9) under 18 or over 85 years; (10) unable to complete clinical evaluation and laboratory measurement for tryptophan and kynurenine; and (11) unavailable for informed consent.

Qualitative analyses for tryptophan and kynurenine concentrations

Liquid chromatography-mass spectrometry (HPLC-MS) was used to measure the serum tryptophan and kynurenine concentrations. Protein precipitation was performed on 100 µL serum samples, and a 10 µL aliquot of the resulting supernatant was analyzed using HPLC-MS/MS. The separation was carried out on an ultra performance liquid chromatography (UPLC) system with a C-18 column using gradient elution. The elution program consisted of acetonitrile (Eluent A) and water with ammonium formate buffer (20 mM, pH 3.7) (Eluent B). The gradient elution conditions were as follows: 0 min = 15% B, 2 min = 15% B, 9 min = 98% B, 11 min = 98% B, 11.5 min = 15% B, and 14 min = 15% B. Prior to injecting the next sample, the column was equilibrated with the initial mobile phase for 5 min. The flow rate was maintained at 0.4 mL/min, with the column temperature set at 50 ℃, and analysis was performed using the 5500 QTRAP (Framingham, MA: AB Sciex Pte. Ltd.) in positive switch mode. The electrospray ionization (ESI) source conditions were as follows: source temperature: 550 ℃; ion source gas1: 55 psi; ion source gas2: 55 psi; curtain gas: 40 psi; and ion spray voltage floating: +4500 V. The multiple reaction monitoring (MRM) method was used for mass spectrometry quantitative data acquisition.

Statistical methods

Continuous variables were presented as mean±standard deviation (SD) or median (interquartile range {IQR}), while categorical variables were described using frequencies and percentages. The normality of continuous data was assessed using the Shapiro-Wilk test and visual inspection of histograms. As the key variables of interest (including serum tryptophan, kynurenine, KTR, and HDS-6 scores) deviated from a normal distribution, non-parametric tests were employed. For comparisons between two independent groups, the Mann-Whitney U test was used. For multiple group comparisons, the Friedman test was used, followed by post-hoc pairwise comparisons using the Wilcoxon signed-rank test with Bonferroni correction (p<0.05 indicating significance). Spearman’s correlation coefficients were used to examine the relationships between serum tryptophan, kynurenine, KTR, and HDS-6 scores. Univariate logistic regression analyses were conducted to assess the individual associations between potential clinical characteristics (e.g., age, gender, smoking status, comorbid conditions) and depressive symptoms (defined by HDS-6 score ≥7). Odds ratios (OR) and 95% confidence intervals (CIs) were calculated. Multivariate logistic regression analyses were performed to evaluate the independent associations between serum tryptophan levels, kynurenine levels, and KTR with depressive symptoms, adjusting for potential confounders (serum albumin and diabetes status). P<0.05 was considered statistically significant. All analyses were conducted using SPSS software version 26.0 (Armonk, NY: IBM Corp.).

## Results

Clinical characteristics of study subjects

Among the included 102 COPD patients, 66 (64.7%) were male, with a median age of 71 years, and 84 (82.4%) were aged 60 years or older. Of these, 44 (43.1%) met the depressive symptom criteria according to the HDS-6 score. Compared to controls, depressed patients had a higher proportion of diabetes; however, there were no statistically significant differences in other demographic characteristics, smoking status, laboratory indicators, and comorbid conditions (Table 1).

**Table 1:** Clinical characteristics of the study participants. COPD: chronic obstructive pulmonary disease

Variables	Control (n=58)	Depression (n=44)	Total (n=102)
Age (years) (mean±SD)	68.8±10.0	70.7±8.0	69.6±9.2
Aged 60 years or over, n (%)	45 (77.6)	39 (88.7)	84 (82.4)
Male, n (%)	37 (63.8)	29 (65.9)	66 (64.7)
Present smoking, n (%)	28 (56.0)	22 (44.0)	50 (49.0)
severe COPD, n (%)	30 (51.7)	28 (63.6)	58 (56.9)
Serum albumin (g/L)	38.1±5.4	40.0±5.6	39.0±5.5
C-reactive protein (mg/L), median (IQR)	5.1 (2.5, 26.4)	3.3 (1.2, 19.8)	4.6 (1.9, 24.8)
Diabetes, n (%)	6 (10.3)	13 (29.6)	19 (18.6)
Heart failure, n (%)	47 (81.0)	39 (88.6)	86 (84.3)
Chronic kidney disease, n (%)	2 (3.4)	2 (4.5)	4 (3.9)

Markers of tryptophan-kynurenine metabolism

**Figure 1:**
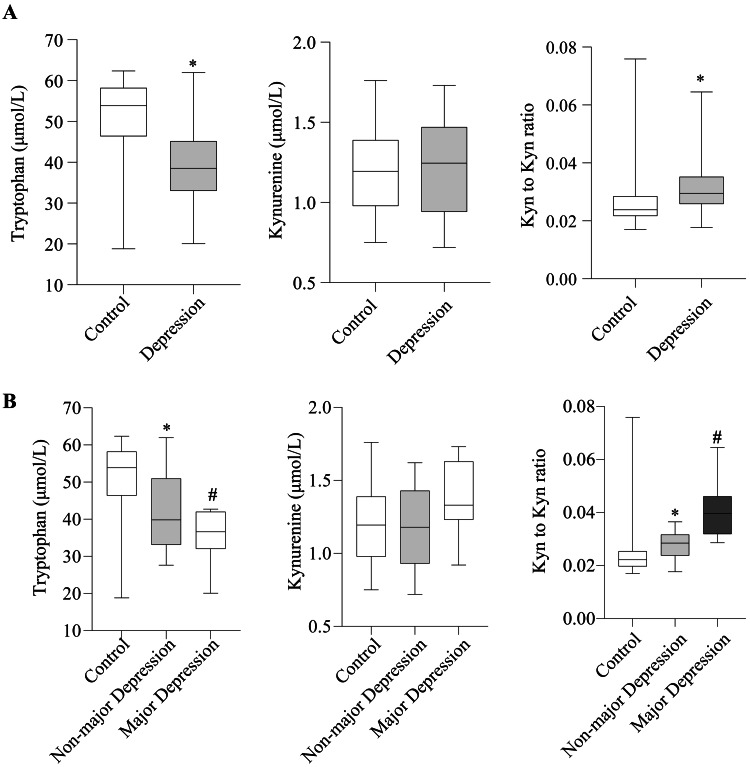
Serum levels of tryptophan, kynurenine, and KTR among different groups. *P<0.05 indicates a significant difference compared to controls. #P<0.05 indicates a significant difference compared to severe depression. (A) Control vs. depression, p-values derived from Mann-Whitney U test; (B) Control vs. non-severe depression vs. severe depression, p-values derived from post-hoc pairwise comparisons with Bonferroni correction. Boxes represent median and interquartile range. KTR: kynurenine-to-tryptophan ratio

**Figure 2:**
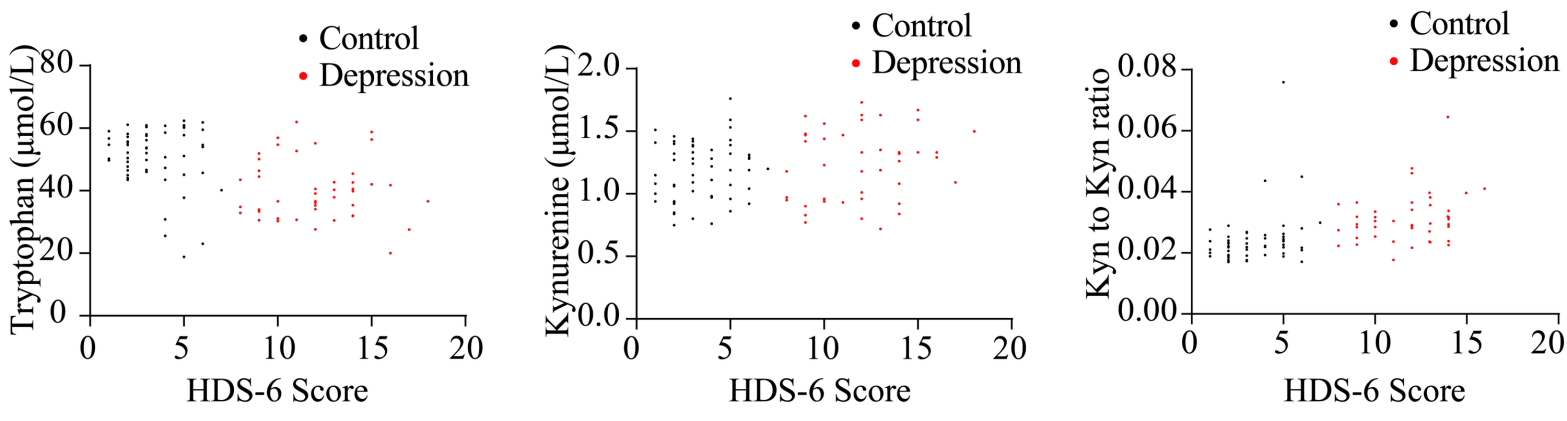
Correlation of HDS-6 score and tryptophan metabolism. Scatter plots reveal a negative correlation between HDS-6 scores and tryptophan levels (Spearman's ρ: -0.478, p<0.001), and positive correlations with KTR (Spearman's ρ: 0.613, p<0.001). However, there was no correlation between HDS-6 scores and serum kynurenine levels (Spearman's ρ: 0.132, p=0.186). HDS-6: six-item Hamilton Depression scale; KTR: kynurenine-to-tryptophan ratio

Compared to the controls, the depressed patients had lower serum levels of tryptophan and a higher kynurenine-to-tryptophan ratio (KTR) (Figure 1A and appendix 1). For patients with severe depressive symptoms, based on an HDS-6 score of 10 or above, the changes in tryptophan and KTR were more pronounced (Figure 1B and appendix 1). Further quantitative correlation analysis was then conducted between serum levels of tryptophan, kynurenine, and KTR with the HDS-6 scores. The results indicated that the degree of dysregulation in the tryptophan-kynurenine metabolism was associated with the severity of depressive symptoms; the greater the severity of depressive symptoms, the lower the levels of tryptophan and the higher the KTR, suggesting an association between depressive symptoms and the dysregulated metabolism of tryptophan along the kynurenine pathway (Figure 2 and appendix 2).

Association of tryptophan-kynurenine metabolism and depressive symptoms

To elucidate the relationship between depressive symptoms and the dysregulated metabolism of tryptophan along the kynurenine pathway, we conducted both univariate and multivariate logistic analyses in a stepwise manner. The univariate logistic analysis revealed that diabetes comorbidity, serum levels of tryptophan, and KTR were significantly associated with depressive symptoms. Additionally, serum albumin levels exhibited a borderline association with depressive symptoms, with a p-value of 0.087 (Table 2).

**Table 2:** Potential clinical characteristics associated with depressive symptom. Potential clinical characteristics associated with depressive symptoms in univariate logistic regression. Depressive symptom was classified by seven points or more on the six-item Hamilton Depression subscale score. COPD: chronic obstructive pulmonary disease; KTR: kynurenine-to-tryptophan ratio

Variables	Odds ratio	Odds ratio (95% confidence interval)	p-Value
Age (every 10 years)	1.255	0.810-1.943	0.309
Female (compared to male)	0.911	0.401-2.073	0.825
Present smoking	0.933	0.426-2.044	0.863
severe COPD	0.612	0.275-1.365	0.230
Serum albumin (every 5 g/L)	1.417	0.951-2.112	0.087
C-reactive protein >10 mg/L	0.872	0.387-1.966	0.741
Diabetes (yes or no)	3.634	1.253-10.539	0.018
Heart failure (yes or no)	0.548	0.175-1.711	0.300
Chronic kidney disease (yes or no)	1.333	0.180-9.856	0.778
Tryptophan level			
Quartile 1 (n=25)	Reference	-	-
Quartile 2 (n=26)	0.222	0.059-0.830	0.025
Quartile 3 (n=26)	0.045	0.011-0.193	<0.001
Quartile 4 (n=25)	0.036	0.008-0.165	<0.001
Kynurenine level			
Quartile 1 (n=25)	Reference	-	-
Quartile 2 (n=26)	0.410	0.131-1.288	0.127
Quartile 3 (n=25)	0.519	0.167-1.611	0.519
Quartile 4 (n=26)	1.077	0.358-3.235	0.895
KTR			
Quartile 1 (n=26)	Reference	-	-
Quartile 2 (n=25)	4.667	0.864-25.193	0.073
Quartile 3 (n=26)	14.000	2.728-71.858	0.002
Quartile 4 (n=25)	63.000	10.460-379.444	<0.001

Subsequently, diabetes status and serum albumin levels were incorporated into multivariate logistic models alongside either serum tryptophan quartiles, kynurenine quartiles, or KTR quartiles. Consistent with the trends observed in the univariate analysis, higher serum tryptophan quartiles were associated with a decreased risk of depressive symptoms, whereas higher KTR quartiles were correlated with an increased risk (Table 3).

**Table 3:** Independent association between depressive symptom and tryptophan metabolism. Association of depression with concentration of tryptophan, kynurenine, and KTR in respective multivariate logistic regression models. Depressive symptom was classified by a score of seven points or more on the six-item Hamilton Depression subscale score. KTR: kynurenine-to-tryptophan ratio

Variables	Odds ratio	Odds ratio (95% confidence interval)	p-Value
Model 1 for tryptophan			
Serum albumin (every increase of 5 g/L)	1.069	0.971-1.178	0.173
Diabetes (yes or no)	54.298	5.740-513.657	<0.001
Tryptophan level			
Quartile 1 (n=25)	Reference	-	-
Quartile 2 (n=26)	0.205	0.052-0.805	0.023
Quartile 3 (n=26)	0.005	0.000-0.065	<0.001
Quartile 4 (n=25)	0.006	0.001-0.067	<0.001
Model 2 for kynurenine			
Serum albumin (every increase of 5 g/L)	1.075	0.988-1.170	0.094
Diabetes (yes or no)	3.621	1.155-11.347	0.027
Kynurenine level			
Quartile 1 (n=25)	Reference	-	-
Quartile 2 (n=26)	0.393	0.119-1.301	0.126
Quartile 3 (n=25)	0.545	0.170-1.751	0.308
Quartile 4 (n=26)	0.758	0.234-2.449	0.643
Model 3 for KTR			
Serum albumin (every increase of 5 g/L)	1.031	0.935-1.136	0.542
Diabetes (yes or no)	8.666	2.264-33.170	0.002
KTR			
Quartile 1 (n=26)	Reference	-	-
Quartile 2 (n=25)	4.605	0.726-29.233	0.105
Quartile 3 (n=26)	12.856	2.166-76.283	0.005
Quartile 4 (n=25)	101.509	14.033-734.257	<0.001

## Discussion

This study reveals significantly reduced serum tryptophan levels alongside increased KTR among COPD patients suffering from depressive symptoms, with more pronounced alterations in severe cases (Figures 1A, 1B). Serum tryptophan levels and KTR were correlated with the HDS-6 scores (Figure 2). In multivariate logistic regression models, higher tryptophan quartiles were associated with a lower risk of depressive symptoms, while higher KTR quartiles increased the risk of depressive symptoms (Table 3). These findings suggest that indices of the tryptophan-kynurenine pathway could serve as potential biomarkers for the assessment of depressive symptoms.

In this study, the detection rate of depressive symptoms was 43.1%, as assessed by the HDS-6 score, which is consistent with previous reports in COPD populations [4,16]. The high incidence of depressive symptoms may partly contribute to the predominance of the elderly in the COPD population. Elderly individuals are more susceptible to depressive symptoms, and the incidence of treatment-resistant depressive symptoms is high [17].

Our findings strengthen the link between dysregulated serum tryptophan-kynurenine metabolism and depressive symptoms in patients with COPD, revealing a clear gradient; greater metabolic disturbance, marked by lower tryptophan and higher KTR, was observed in those with more severe depressive symptoms. This study appears to be the first investigation specifically examining this relationship in the context of COPD. Given the established role of chronic inflammation in COPD, the results of this support the hypothesis that the systemic inflammatory state may aggravate tryptophan-kynurenine pathway dysregulation, thereby contributing to depressive symptomatology [14,15]. This notion is consistent with previous research; for instance, interferon-α treatment, known to induce inflammatory responses, has been shown to decrease tryptophan, increase kynurenine and KTR, and promote depressive symptoms [18]. Similarly, a meta-analysis confirmed that immune activation in chronic diseases is associated with tryptophan-kynurenine imbalance and subsequent depression [14]. Together, these findings underscore inflammation as a potential mediator between tryptophan metabolism dysregulation and depressive symptoms in COPD.

The observed association between peripheral tryptophan levels, their metabolites, and depressive symptoms is likely attributable to the role of the tryptophan-kynurenine pathway in mood regulation. Patients with depressive disorders often exhibit insufficient serotonin (5-HT) function in the brain [8]. Tryptophan is a precursor for the biosynthesis of 5-HT, and changes in its levels within the brain can lead to alterations in 5-HT content [18]. Studies in both humans and animals have confirmed that acute tryptophan depletion can induce depressive symptoms, while supplementation with tryptophan can help improve these symptoms [19]. Although our study does not directly address whether central tryptophan metabolism along the kynurenine pathway mediates depression, our findings suggest that these peripheral indicators, such as levels of peripheral tryptophan and its metabolites, can serve as potential biomarkers for the assessment of depressive symptoms, possibly through their involvement in the tryptophan-kynurenine pathway, which is a major route of tryptophan metabolism. Given that central markers, such as cerebrospinal fluid testing, are not routinely performed, our results provide a complementary approach to identifying depressive symptoms.

The association between low tryptophan levels and depressive symptoms also suggests that tryptophan supplementation may represent an alternative strategy for antidepressant treatment. Prior to 1990, tryptophan was widely used as an adjunctive therapy for clinical conditions, such as insomnia, anxiety, mood depression, and premenstrual syndrome, with a long history of use in the United States [20]. The U.S. Food and Drug Administration also designated it as a dietary supplement. However, at that time, the appropriate dosage of tryptophan required by the human body was unknown, and extremely high doses were administered. Due to outbreaks of eosinophilia-myalgia syndrome related to tryptophan supplementation, this therapy was later discontinued. Although it is now clear that low-dose tryptophan supplementation is safe, past experiences have made the indications for choosing tryptophan supplementation more rigorous [20]. Limited by the lack of widespread testing for tryptophan, it is not easy to screen for individuals with tryptophan metabolism disorders; thus, this approach has not become popular again [21]. In recent years, with the development of metabolomics research, tryptophan testing has become much easier [22]. The relationship between tryptophan metabolism disorders and depression and sleep disorders has once again garnered attention [18,23]. Therefore, elucidating the tryptophan metabolism in COPD-related depressive symptoms aids in a better understanding of the pathogenesis of this mental disorder and in exploring therapeutic strategies. Given the growing body of evidence linking tryptophan metabolism disorders to depression in various clinical populations, it is valuable to further investigate whether tryptophan supplementation could be a beneficial intervention for depression associated with hypotryptophanemia [18].

To minimize factors that could potentially interfere with peripheral levels of tryptophan and its metabolites, and to investigate depression, this study excluded patients with potential infections, metabolic or nutritional disorders, as well as those who could not accurately assess their depressive symptoms. This inevitably introduced a certain degree of selection bias. However, this cross-sectional study employed a consecutive sampling technique, enrolling all eligible patients during the study period. This method reduces selection bias by minimizing the researcher's discretion in patient selection and providing a more representative sample of the severe COPD population. Owing to the homogeneity of the included sample, the results of this study cannot be directly generalized to all COPD populations. Additionally, the small sample size limited us from performing subgroup analyses; thus, we could not explore the diagnostic performance of tryptophan and KTR and their potential reference ranges. These limitations await further exploration in future studies with larger sample sizes.

Current diagnostic methods for depressive symptoms in COPD patients rely heavily on clinical symptoms and self-reported measures, which can be subjective and prone to bias [3,16]. Thus, the identification of reliable biomarkers for COPD-related depressive symptoms is crucial for improving diagnostic accuracy and treatment outcomes. Objective biomarkers, such as serum levels of tryptophan metabolites along the kynurenine pathway, could enhance the precision of depressive symptom diagnosis in COPD patients. Moreover, understanding the role of tryptophan metabolism in COPD-related depression may open new avenues for therapeutic strategies. Interventions aimed at modulating the kynurenine pathway, such as dietary modifications or pharmacological agents, could potentially alleviate depressive symptoms in COPD patients. Additionally, these biomarkers could be used to monitor treatment response and adjust therapies accordingly, ultimately improving patient outcomes and quality of life.

This study has some limitations. We did not comprehensively analyze the entire tryptophan-kynurenine metabolic pathway, including metabolites such as kynurenic acid, quinolinic acid, and 3-hydroxykynurenine, which could offer deeper insights into the pathogenesis of depression [9,10]. Furthermore, although we excluded patients on recent glucocorticoid therapy, we did not systematically adjust for the potential effects of other medications, such as common COPD inhalers, which may represent another source of unmeasured confounding. Additionally, beyond pharmacological factors, we could not fully elucidate the potential confounding effects of several other conditions associated with depression on tryptophan metabolism. For instance, changes in the kynurenine pathway have been linked to cognitive decline and depression in aging populations [24], and dysregulation in tryptophan metabolism has been observed in post-stroke patients, which may contribute to the high incidence of depression [25]. Since this study population was predominantly elderly COPD patients and none had stroke-related conditions (data not shown), we were unable to assess the impact of these conditions on tryptophan levels (Table 1). More broadly, the presence of various comorbid conditions known to influence tryptophan-kynurenine metabolism, such as diabetes and heart failure, in our real-world COPD cohort, while increasing the generalizability of our findings to clinical practice, may affect the reproducibility of our findings in populations with different comorbidity profiles [26]. Future studies in larger and more diverse COPD cohorts should conduct a more thorough analysis of the tryptophan-kynurenine metabolites and their correlation with depressive symptoms, and compare the cost-effectiveness of measuring serum tryptophan and KTR vs. the entire pathway. Future research should also explore interventions targeting this pathway, such as dietary modifications, pharmacological agents, or probiotics, to offer novel therapeutic options [27]. Additionally, mechanistic studies are needed to elucidate the interactions between inflammation, tryptophan metabolism, and depression in COPD [14]. If validated, serum tryptophan and KTR could serve as objective biomarkers to improve diagnosis, guide treatment decisions, and monitor treatment response, ultimately enhancing patient outcomes. Finally, aside from the tryptophan-kynurenine pathway, other unmeasured metabolic factors could potentially confound the observed associations, and our findings should be interpreted within the context of the variables we were able to account for. Fourth, while we recognized the potential influence of nutritional status on tryptophan metabolism and adjusted for serum albumin levels in our multivariate models, the absence of body mass index (BMI) data is a limitation. Although serum albumin is an objective marker, BMI provides complementary information on energy reserves and body composition. Therefore, we cannot rule out residual confounding by overall nutritional status. Future studies should include anthropometric measurements to better account for this aspect.

## Conclusions

Our results suggest that tryptophan metabolism along the kynurenine pathway may be involved in the pathogenesis of depressive symptoms in COPD populations, and serum tryptophan levels and KTR could serve as potential markers for assessing depressive symptoms. Future research should focus on validating these biomarkers in larger, diverse cohorts and exploring interventions that target this metabolic pathway to alleviate depressive symptoms in COPD.

## Appendices

Appendix 1

**Table 4:** Comparison of tryptophan-kynurenine pathway markers between groups. P-values derived from Mann-Whitney U test (A) or post-hoc pairwise comparisons with Bonferroni correction (B). KTR: kynurenine-to-tryptophan ratio

Groups	n	Tryptophan, median (IQR)	Kynurenine, median (IQR)	KTR, median (IQR)	p-Value (vs. control)
A. All participants					
Control	58	53.8 (46.4, 58.2)	1.20 (1.00, 1.39)	0.022 (0.020, 0.0.025)	<0.05
Depressive symptoms	44	38.5 (33.0, 45.2)	1.24 (0.94, 1.47)	0.030 (0.026, 0.035)	
B. By severity					
Control	58	53.8 (46.4, 58.2)	1.20 (1.00, 1.39)	0.022 (0.020, 0.0.025)	<0.001
Non-major depressive symptoms	33	39.8 (33.1, 51.0)	1.18 (0.93, 1.43)	0.028 (0.024, 0.032)	
Major depressive symptoms	11	36.6 (32.0, 42.0)	1.13 (1.22, 1.63)	0.040 (0.032, 0.046)	

Appendix 2 

**Table 5:** Spearman's correlation between HDS-6 scores and tryptophan-kynurenine pathway markers. HDS-6: six-item Hamilton Depression scale; KTR: kynurenine-to-tryptophan ratio

Variables	Spearman's ρ	p-Value
Tryptophan	-0.478	<0.001
Kynurenine	0.132	0.186
KTR	0.613	<0.001

## References

[1] Vogelmeier CF, Criner GJ, Martinez FJ (2017). Global strategy for the diagnosis, management, and prevention of chronic obstructive lung disease 2017 report. GOLD executive summary. Am J Respir Crit Care Med.

[2] Yin P, Wu J, Wang L (2022). The burden of COPD in China and its provinces: findings from the Global Burden of Disease Study 2019. Front Public Health.

[3] Yohannes AM, Alexopoulos GS (2014). Depression and anxiety in patients with COPD. Eur Respir Rev.

[4] Martínez-Gestoso S, García-Sanz MT, Carreira JM (2022). Impact of anxiety and depression on the prognosis of copd exacerbations. BMC Pulm Med.

[5] Moncrieff J, Cooper RE, Stockmann T, Amendola S, Hengartner MP, Horowitz MA (2023). The serotonin theory of depression: a systematic umbrella review of the evidence. Mol Psychiatry.

[6] Kendrick T, Collinson S (2022). Antidepressants and the serotonin hypothesis of depression. BMJ.

[7] Almulla AF, Maes M (2023). Although serotonin is not a major player in depression, its precursor is. Mol Psychiatry.

[8] Adrien J (2002). Neurobiological bases for the relation between sleep and depression. Sleep Med Rev.

[9] Chen X, Xu D, Yu J, Song XJ, Li X, Cui YL (2024). Tryptophan metabolism disorder-triggered diseases, mechanisms, and therapeutic strategies: a scientometric review. Nutrients.

[10] Marx W, McGuinness AJ, Rocks T (2021). The kynurenine pathway in major depressive disorder, bipolar disorder, and schizophrenia: a meta-analysis of 101 studies. Mol Psychiatry.

[11] Chen LM, Bao CH, Wu Y (2021). Tryptophan-kynurenine metabolism: a link between the gut and brain for depression in inflammatory bowel disease. J Neuroinflammation.

[12] Lodge KM, Vassallo A, Liu B (2022). Hypoxia increases the potential for neutrophil-mediated endothelial damage in chronic obstructive pulmonary disease. Am J Respir Crit Care Med.

[13] Rabe KF, Rennard S, Martinez FJ (2023). Targeting type 2 inflammation and epithelial alarmins in chronic obstructive pulmonary disease: a biologics outlook. Am J Respir Crit Care Med.

[14] Hunt C, Macedo E Cordeiro T, Suchting R (2020). Effect of immune activation on the kynurenine pathway and depression symptoms - a systematic review and meta-analysis. Neurosci Biobehav Rev.

[15] Torosyan R, Huang S, Bommi PV (2021). Hypoxic preconditioning protects against ischemic kidney injury through the IDO1/kynurenine pathway. Cell Rep.

[16] Stage KB, Middelboe T, Pisinger C (2003). Measurement of depression in patients with chronic obstructive pulmonary disease (COPD). Nord J Psychiatry.

[17] Cappon D, den Boer T, Jordan C, Yu W, Metzger E, Pascual-Leone A (2022). Transcranial magnetic stimulation (TMS) for geriatric depression. Ageing Res Rev.

[18] Correia AS, Vale N (2022). Tryptophan metabolism in depression: a narrative review with a focus on serotonin and kynurenine pathways. Int J Mol Sci.

[19] Russo S, Kema IP, Fokkema MR (2003). Tryptophan as a link between psychopathology and somatic states. Psychosom Med.

[20] Kaufman LD, Philen RM (1993). Tryptophan: current status and future trends for oral administration. Drug Saf.

[21] Xiao C, Chen Y, Liang X (2014). A modified HPLC method improves the simultaneous determination of plasma kynurenine and tryptophan concentrations in patients following maintenance hemodialysis. Exp Ther Med.

[22] Wang LS, Zhang MD, Tao X (2019). LC-MS/MS-based quantification of tryptophan metabolites and neurotransmitters in the serum and brain of mice. J Chromatogr B Analyt Technol Biomed Life Sci.

[23] Shaw K, Turner J, Del Mar C (2002). Tryptophan and 5-hydroxytryptophan for depression. Cochrane Database Syst Rev.

[24] Davidson M, Rashidi N, Nurgali K, Apostolopoulos V (2022). The role of tryptophan metabolites in neuropsychiatric disorders. Int J Mol Sci.

[25] Liu L, Wu Z, Lu Y, Lu W, Su G, Zhou Z (2024). Effects of phototherapy on biopterin, neopterin, tryptophan, and behavioral neuroinflammatory reaction in patients with post-stroke depression. Sci Rep.

[26] Gabela AM, Mthembu N, Hadebe S (2025). Tryptophan metabolism in health and disease- implications for non-communicable diseases. Immunol Lett.

[27] Rudzki L, Ostrowska L, Pawlak D, Małus A, Pawlak K, Waszkiewicz N, Szulc A (2019). Probiotic Lactobacillus plantarum 299v decreases kynurenine concentration and improves cognitive functions in patients with major depression: a double-blind, randomized, placebo controlled study. Psychoneuroendocrinology.

